# Genetic trends in maternal and neonatal behaviors and their association with perinatal survival in French Large White swine

**DOI:** 10.3389/fgene.2014.00410

**Published:** 2014-12-02

**Authors:** Laurianne Canario, Jean-Pierre Bidanel, Lotta Rydhmer

**Affiliations:** ^1^Institut National de la Recherche Agronomique, Unité Mixte de Recherche 1388, Génétique, Physiologie et Système d'ElevageCastanet-Tolosan, France; ^2^Institut National de la Recherche Agronomique, Unité Mixte de Recherche 1313, Génétique Animale et Biologie IntégrativeJouy-en-Josas, France; ^3^Department of Animal Breeding and Genetics, Swedish University of Agricultural SciencesUppsala, Sweden

**Keywords:** maternal behavior, piglet vitality, farrowing, genetic trend, sow

## Abstract

Genetic trends in maternal abilities were studied in French Large White sows. Two lines representing old-type and modern-type pigs were obtained by inseminating modern sows with semen from boars born in 1977 or 1998. Successive generations were produced by inter-se mating. The maternal performance of sows from the second generation was compared in farrowing crates. Video analysis was performed for the 1st h after the onset of 43 and 36 farrowing events, and for the 6 first hours for 23 and 21 events, in old-type and modern-type sows, respectively. Genetic trends were estimated as twice the difference in estimates between the 2 lines. The contribution of behavior to the probability of stillbirth and piglet death in the first 2 days was estimated as the percentage of deviance reduction (DR) due to the addition of behavior traits as factors in the mortality model. Sow activity decreased strongly from the 1st to the 2nd h in both lines (*P* < 0.001). In the first 6 h, old-type sows sat (1st parity), stood (2nd parity) and rooted (both parities) for longer than modern-type sows, which were less active, especially in 2nd parity. In modern-type sows, stillbirth was associated positively with lying laterally in the first 6 h (4.6% DR) and negatively in the 1st h (9.1% DR). First-parity old-type sows were more attentive to piglets (*P* = 0.003) than modern-type sows which responded more to nose contacts at 2nd parity (*P* = 0.01). Maternal reactivity of modern-type sows was associated with a higher risk of piglet death (4.6% DR). Respiratory distress at birth tended to be higher in modern-type piglets than in old-type piglets (*P* < 0.10) and was associated with a higher risk of piglet death in both lines (2.7–3.1% DR). Mobility at birth was lower in modern-type than old-type piglets (*P* < 0.0001). Genetic trends show that sow and piglet behaviors at farrowing have changed. Our results indicate reduced welfare in parturient modern-type sows and their newborn piglets.

## Introduction

Highly productive lean sows are affected by undesirable correlated effects of genetic selection, including modifications of behavior that affect both their own welfare as well as that of their progeny (Rauw et al., 1998; Rauw, 2007; Canario et al., 2013). In particular, a rise in the sensitivity to stressors in the physical environment is observed when intensive genetic selection for a few traits is applied (Grandin and Dessing, 2014). Sows face acute stressors when they endure a sudden change in their environment, such as the critical period of farrowing, especially in primiparous females. In pigs, the survival of progeny depends strongly on maternal care during the first days of life. Sow behavior is a major component of maternal success in terms of piglet survival and growth. Thodberg (2001) suggested that good sow maternal behavior involved limited activity in the peripartum period, but that farrowing should be preceded by a period of nest-building activity (e.g., Thodberg et al., 1999; Damm et al., 2005). At farrowing, such activity can continue although lying laterally and changing posture only infrequently is preferable to reduce the risks of stillbirth and crushing new-born piglets. At the same time, limiting changes in posture allows for easier access to the udder where piglets find warmth and colostrum (Petersen et al., 1990; Jarvis et al., 1999). Paradoxically, the development of proper mother-progeny bonding requires postural changes so that the sow can interact with the piglets (Jarvis et al., 1999; Pedersen et al., 2003).

The evolution of sow maternal behavior in response to domestication has been studied by comparing domestic sows with wild sows or wild boar × domestic sows. No trend was observed for behavior around farrowing (e.g., Jensen, 1986; Jensen et al., 1991; Horrell, 1997).

Genetic selection schemes for lean growth rate and prolificacy conducted in pig dam lines at the end of the last century led to increased piglet mortality around farrowing (Tribout et al., 2003; Canario, 2006). Since then, selective breeding programs have been successfully modified in order to limit stillbirth; however neonatal mortality remains a serious problem. Little is known about the genetic trends in sow maternal behavior associated with genetic selection for both lean growth rate and prolificacy. It is nevertheless reasonable to hypothesize that the production of larger litters requires a higher maternal investment than in the past. In 1977 Bidanel and collaborators initiated an experiment aimed at estimating genetic trends for performance in French Large White (LW) pigs over a 21-year period (1977–1998) corresponding to approximately 11 generations of selection (Tribout et al., 2010). The principle of the experiment was to use frozen semen from boars that were representative of the two populations raised at the beginning and the end of the 21-year period to inseminate modern-type sows and produce 2 lines (Smith, 1977). The next generations were produced by inter-se mating. Animals were compared in the same environment for a large number of traits. The animals from the two lines will be referred to as old-type pigs and modern-type pigs, respectively. Modifications in sow maternal performance were investigated in detail in sows from the second generation and their progeny (Canario, 2006).

The consequences of genetic selection on animal behavior have rarely been investigated, especially for traits that are difficult and time-consuming to record. Based on the above-described experimental design, we estimated whether sow behavior has been modified as a correlated response to selection for lean meat growth and prolificacy in the French Large White population. In this paper, we establish genetic trends for behaviors related to sow farrowing activity, newborn vitality, and their associations with piglet mortality in the first 48 h after birth.

## Materials and methods

### Experimental design

The animals were produced and raised in the INRA experimental herd of Avord (Cher). Sows were managed under a batch farrowing system, with 3 weeks interval between successive batches. Animals were cared for according to the protection of animals rules defined in the French law (Code Rural, articles R214-64–R214-71; http://www.legifrance.gouv.fr). The history of selection over the study period can be summarized as follows: until the mid-1980s, pigs were selected for growth rate, feed efficiency, and carcass leanness; in 1985, a meat quality index was introduced in the breeding goal; at the end of the 1980s, a strong emphasis was placed on improving the litter size through the generalization of so-called “hyperprolific” breeding schemes. Finally, in the mid-1990s, standard selection indexes were replaced by more accurate predictors of breeding values based on multiple-trait BLUP animal model methodology. At this time, the criterion of selection for litter size was the total number of piglets born. Management and other environmental conditions have improved progressively over the 21-year period considered, with, for instance, an increasing knowledge of nutritional requirements of animals and the generalization of artificial insemination.

**Figure 1:**
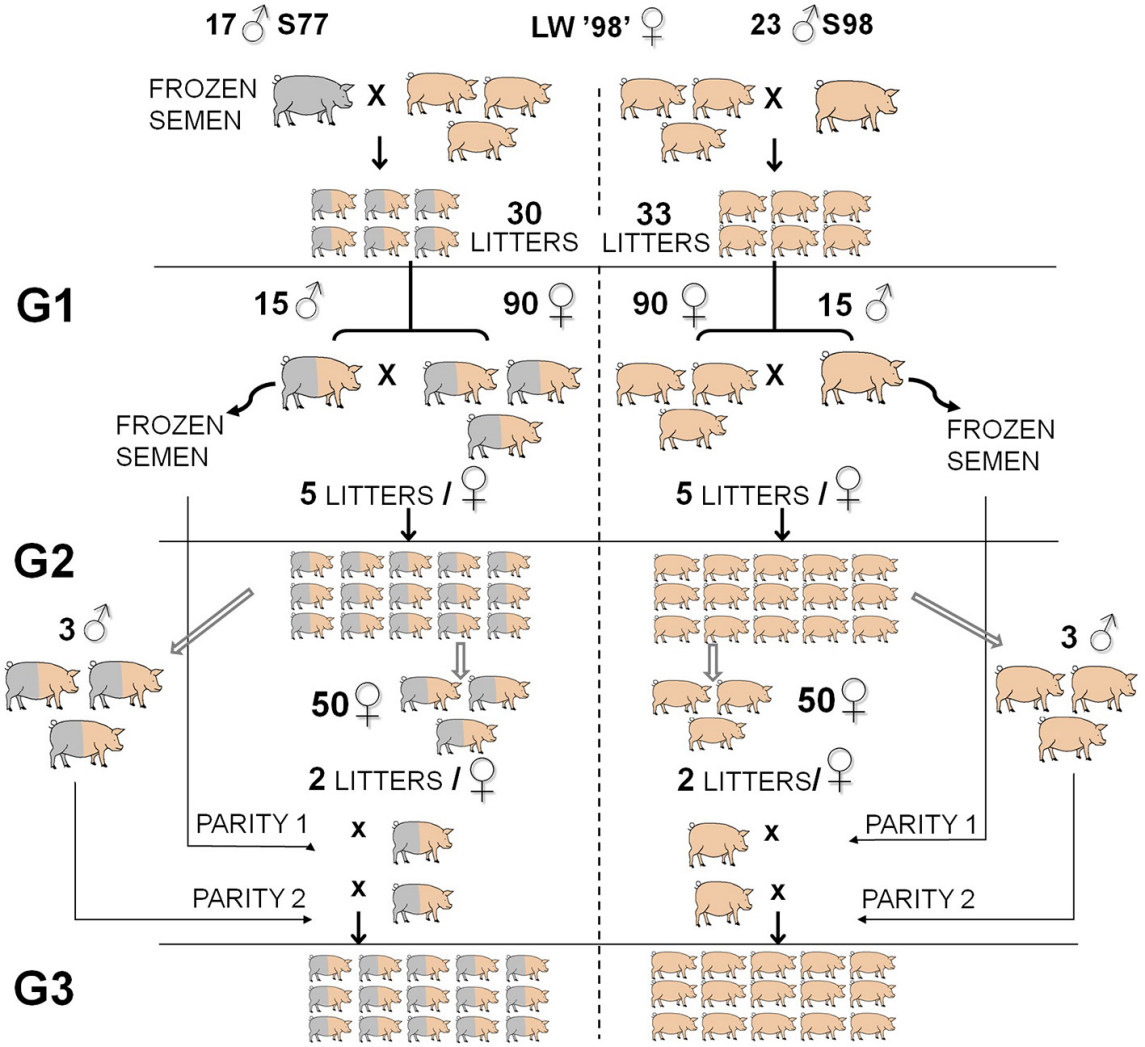
**Overview of the experimental design developed to estimate genetic trends from 1977 to 1998 in the French Large White dam population**. Phenotyping of the maternal performance of old-type and modern-type lines was carried out in the first 2 parities of G2 (second generation) sows and their progeny (G3). Modified from Tribout et al. (2010).

The two lines (referred to as old-type and modern-type, respectively) have been produced by inseminating French LW sows born in 1998 with semen from LW boars born either in 1977 or in 1998 (Tribout et al., 2010; Figure 1). Three generations of old-type and modern-type pigs were then produced by inter se mating of randomly chosen old-type or modern-type boars and gilts. The difference observed between the 2 lines shows half of the genetic change. In the present experiment, sows from the 2nd generation were inseminated twice at a 12-h interval with frozen semen from boars of the first generation in first parity and with fresh semen from boars of the second generation in second parity. The maternal performance of sows and litter characteristics were recorded from August 2003 to September 2004. Sows were managed in a batch-farrowing system, with a 3-weeks interval between successive batches. They were fed 2.5–3 kg of a commercial sow diet twice daily during the whole gestation period. Approximately 1 week before expected date of farrowing, they were moved to one of the 2 farrowing units. Sows were housed in farrowing crates (1.80 × 2.40 m; space available to the sow: 0.60 × 1.90 m) on a partially slatted flooring covered with a thin floor of straw and made with solid external wooden walls (height: 0.5 m) on the four sides, so that sows could see their neighbors. As often as possible, old-type and modern-type sows were placed in neighboring farrowing crates, so that an old-type female had modern-type neighbor females. The room was lit both by natural daylight and artificial lighting maintained all around the clock. Sows were fed a commercial diet twice a day according to regular management practices and had permanent access to water from a nipple drinker. Feed was distributed at 8:00 a.m. and 4:30 p.m. Crates were cleaned daily at 8:00 p.m. Sows were daily provided with 1 kg straw, from 2 days before to 4 days after the date of farrowing, so that they had continuous access to straw during the experimental period. A water nipple for the piglets was also present, as well as a ceramic heat lamp located at the back of the sow until day 3.

From day 111 of gestation, sows were daily visited to identify signs of impending farrowing and to reduce their fear of humans. The farrowing was not induced. Birth assistance including ocytocin treatment and/or vaginal palpations was restricted to cases of extreme necessity and implied the removal of the sow from the study. Care was provided to the sows when essential to respect the general guidelines outlined in the European animal welfare regulations applicable at this time. Farrowing supervision was carried out 24 h a day. Disturbance of the sows was limited by video watching from an adjacent room. Apart from manipulation of newborn piglets that stimulate their vitality, interference with the natural farrowing process was avoided. There was no human intervention to control aggression or prevent crushing of newborn piglets. Cross-fostering was not allowed. Ear marking and tail trimming was performed on day 2 and male piglets were castrated on day 4. The onset of farrowing corresponded to the time of birth of the first piglet. Each expelled piglet was immediately caught. Its umbilical cord was cut and a blood sample taken for plasma parameter measurements. The remaining part of the umbilical cord was ligatured with a surgical silk. Subsequently, the piglet was carried to a weighing place located inside the maternity to be dried with straw and drying paper, weighed, sexed and marked on its back with a number corresponding to its birth order. Next, it was replaced in the back part of the crate, close to the vulva of its dam. Piglets were weaned at 4 weeks of age. A total of 137 litters and 1679 piglets were produced.

### Litter mortality traits

The fine monitoring of stillbirth allowed the number of piglets born alive to be exactly known. Piglet mortality was carefully registered during the first 2 days after birth and the causes of deaths were determined by a macroscopic examination. They were classified in three categories: (1) thin piglets dying with chops palpable or visible under the skin, presumably because of starvation, were classified as weak; (2) piglets dying because of injuries caused by the sow were classified as crushed; and (3) other causes, including unidentified cause, and cannibalism.

### Behavioral traits

Sows from the 2 lines could not be visually differentiated. The onset of farrowing was determined on 61 and 52 farrowing events in old-type and modern-type sows, respectively. Sow and piglet behavior was recorded using 24 time lapse video (VHS Panasonic video recorder associated with DPX9 *multiplexer Advanced Technology Video*). Video tapes were analyzed by a single observer by continuous observations with speeded up watching. Behaviors were analyzed as durations and/or occurrence. Time of birth, time to first contact with the udder and time to first intake of colostrum were recorded for each piglet. First, behavioral analyses at farrowing were limited to a 6 h period beginning with the birth of the first piglet to depict finely the pattern of sow activity in the first hours and to identify the change from a period of high activity—elicited by the onset of farrowing—to a period of lower activity. A total of 23 old-type sows including 8 first and 15 second parity sows, and 21 modern-type sows including 6 first and 15 second parity sows, were compared. The behavioral traits included the sow postural activity, rooting behavior, and attention and responsiveness toward progeny. Rooting behavior was visible only during the first 4 h. The behavioral definitions for sow and piglet measurements are given in Tables 1, 2, respectively. Second, sow postural activity was analyzed during the first hour after the onset of farrowing on 26 first-parity and 17 second-parity old-type sows and 23 first-parity and 13 second-parity modern-type sows.

**Table 1:** **Behavioral measurements for the comparison of old-type sows and modern-type sows**.

**Behavior**	**Definition**
**VIDEO OBSERVATION**	
**Postural activity**	
Lying ventrally	Lying in sternal recumbency, with udder not exposed
Lying laterally	Lying in lateral recumbency, with udder exposed
Sitting	Sitting continuously for at least 5 s
Standing	Standing upright, on four feet
Postural changes	All changes between the four positions mentioned above
**Exploratory activity**	
Rooting	Head making a scooping motion with the nose in contact with the floor (and/or straw) in a scaterring way
**Maternal activity**	
Piglet examination	Movement of the snout toward the approaching piglet, located at less than one piglet length from the sow snout
Piglet indifference	No visible reaction to the approaching piglet, located at less than one piglet length from the sow snout
Piglet responsiveness	Ratio of piglet examinations above trials (sum of piglet examinations and indifferences)
Piglet attentiveness	Head directed attentively to at least one piglet, located at more than one piglet length from the sow snout
**DIRECT OBSERVATION**	
**Reaction to newborn handling**	
Maximal postural change	Sows were in lateral recumbency at the beginning of the observation. The different postures corresponded to lying laterally, lying ventrally, sitting, and standing
Vocalizations	Vocalizations were registered according to the following ordered scale: 0, no grunting; 1, some isolated grunts (*n* < 5); 2, regular grunts; 3, rhythmic high intensity grunts
**Reaction to first nose contact with a newborn**	
Maximal postural change	Same definition as above
Vocalizations	Same definition as above
Investigation	Four ordinal categories: 0, no answer; 1, piglet calm sniffing; 2, piglet strong sniffing; 3, attempt on biting piglet
Sniffing	Piglet calm or strong sniffing
Aggressive reaction	Piglet strong sniffing, or attempt on biting piglet

**Table 2:** **Behavioral measurements for the comparison of old-type and modern-type newborn piglets**.

**Behavior (criterion)**	**Definition**
**VIDEO OBSERVATION**	
**Suckling activity**	
Time to first udder contact (min)	Time interval between birth and first touching of the udder with nose
Time to first colostrum intake (min)	Time interval between birth and immobilization at the udder, holding a teat in mouth with rapid mouth movements for at least 5 s
Activity at the udder (#)	Number of piglets suckling (teat in mouth or massaging the udder actively)
**DIRECT OBSERVATION**	
Respiratory difficulty (0/1)	The piglet shows difficulties to breath normally, makes attempts to breath, with visible exaggerate movements of the mouth
Mobility at birth (class)	Evaluated at the birth weighing: the piglet 0, doesn't move at all; 1, shows some movements; 2, shows many movements to stand up or even stand up
Vocalizations at birth (class)	Evaluated at the birth weighing: the piglet 0, doesn't scream; 1, makes few vocalizations; 2, makes many vocalizations

On-field behavioral observations were also realized. Several reactions of the sow were registered by direct observations when animals were manipulated by humans. The 3 observers had trained together in preliminary trials to register the behavioral items in similar way. The behavioral reaction of the sow to first handling of a newborn piglet and at the first nose contact with a newborn piglet was quantified via postural changes and vocalizations. The catch up of piglets was a rapid action without staying stationary at the back of the crate. The initial posture of the sow was recorded. Once the piglet was taken out from the crate, the sow maximum posture reached and vocalizations were recorded. Piglet vitality at birth was assessed through direct observation of the individual difficulty to breath, mobility, and intensity of vocalizations while weighed in a standard box (60 × 40 × 35 cm^3^).

### Statistical comparison of the lines

Statistical analyses were performed using the Statistical Analysis System Software (SAS Institute, Inc.). Stillbirth and early mortality traits were analyzed as raw values and as the percentage of piglets born in total and born alive, respectively. If normally distributed, behavior traits were analyzed with the MIXED procedure. When binomially or Poisson distributed, they were analyzed using the GEE option from the SAS GENMOD procedure.

The general model for analyses of sow traits included the fixed effects of farrowing batch, line, parity, and the line × parity interaction, plus a sow random effect. In addition, a fixed effect of the observer was included for sow reactivity when recorded on farm. For the video data corresponding to the first hour from 79 farrowing events and the 6 first hours from 44 farrowing events, line differences were first estimated globally over the whole period of time using the model described above. Next, for the description of the farrowing pattern, analyses were carried out on a per hour basis. The model included in addition to the previous model the fixed effects of the Period of Time (PT = first to 6th h after onset of farrowing), the line × PT and parity × PT interactions. When not significant (*P* > 0.10), interactions were removed from the model. The covariance between measurements at different time intervals within the same sow was allowed to vary according to an exchangeable structure. Then, patterns of line × PT behaviors were drawn.

As regards to piglet traits, respiratory difficulty and reaction at birth were considered as binomially and Poisson distributed, respectively. The model included the fixed effects of farrowing batch, line, parity, line × parity interaction plus the random effect of the litter of birth. Udder activity was recorded on 12 litters in each line among which 11 old-type piglets and 21 modern-type piglets had no time record for the first contact with the udder and 18 piglets and 32 modern-type piglets had no time record for the first intake of colostrum. Two situations occurred: (1) these pigs did not suckle and therefore observation periods became extremely long. In such a case, they were attributed the value of 3 h; (2) in large litters, it became more and more difficult to see individual piglets reaching the udder while the number of born piglet increased. Time to first udder contact and first intake of colostrum was analyzed with a model including the fixed effects of the line, farrowing batch and a random effect of the litter of birth.

Estimates are given after a back transformation to the original scale: when having a Poisson distribution, results on the original scale were obtained via an exponential transformation and when binomially distributed, results were obtained via an exp(y)/(1 + exp(y)) transformation where y was the least square means estimate on the logit scale. The realized genetic trends from 1977 to 1998 (ΔG) and their standard errors (SE(ΔG)) were estimated for each trait as proposed by Smith (1977): ΔG = 2 × (modern-type lsmean—old-type lsmean) and SE(ΔG) = 2 × SE(modern-type lsmean—old-type lsmean).

### Relationship between behavior and piglet mortality

**Figure 2:**
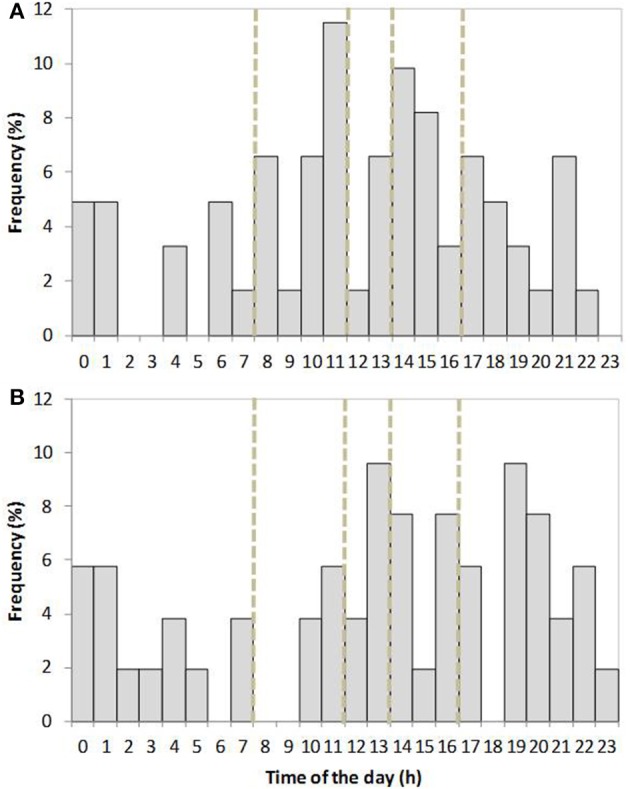
**Distribution of the onset of farrowing over the circadian period in old-type (A) and modern-type (B) sows**. Periods of staff working hours are indicated with doted lines (8–12 a.m. and 2–5 p.m.), data from the first and second parities are grouped together.

**Figure 3:**
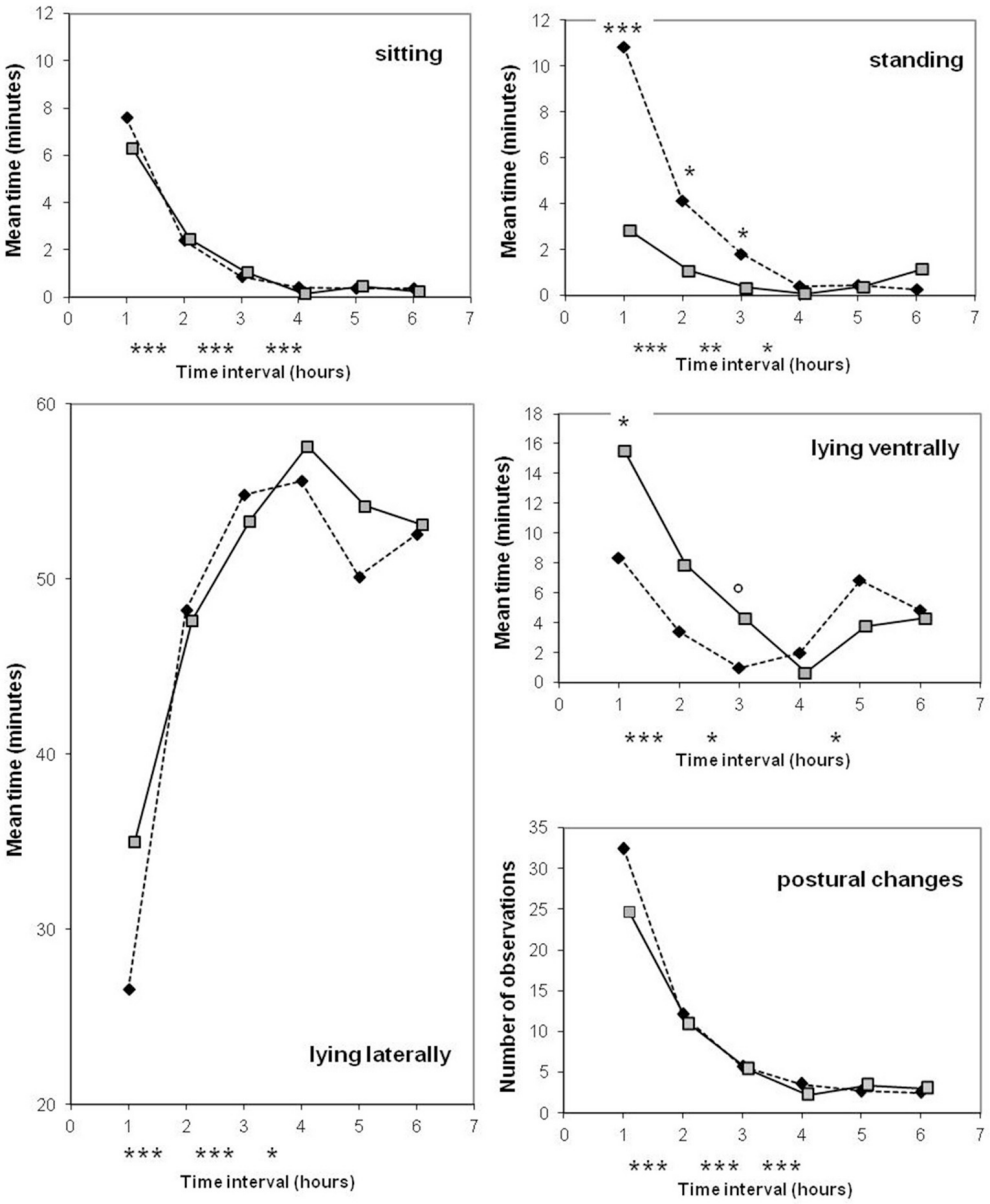
**Comparison of old-type sows (♦) and modern-type sows (**
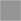
**) for postural activity during the first 6 h after the onset of farrowing**. Significance levels below the X axis refer to differences between successive periods of time, obtained with both lines merged together. Significance levels reported on the graph above mean values refer to lines differences: ^◦^*P* < 0.10; ^*^P < 0.05; ^**^P < 0.01; ^***^P < 0.001.

**Figure 4:**
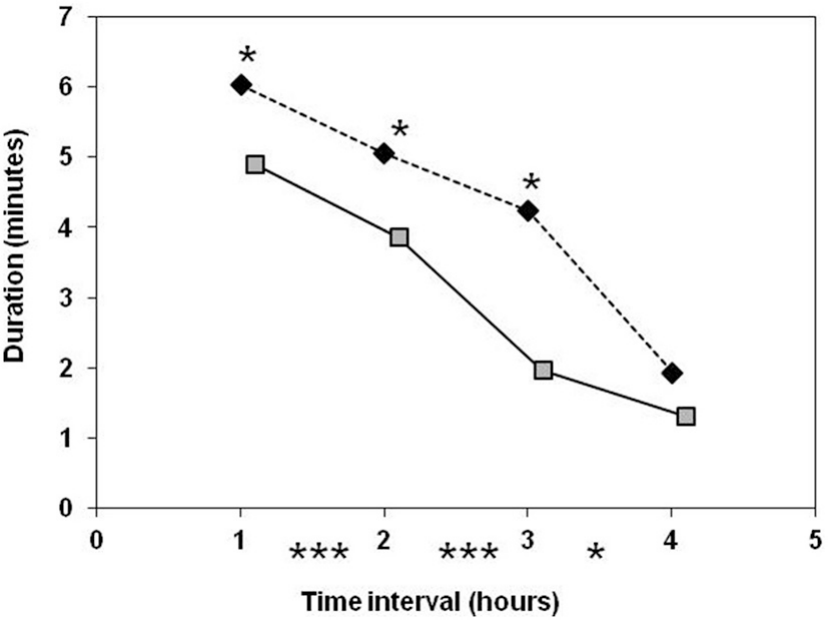
**Comparison of old-type sows (♦) and modern-type sows (**
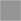
**) for rooting activity during the first 4 h after the onset of farrowing**. Significance levels below the X axis refer to differences between successive periods of time, obtained with both lines merged together. Significance levels reported on the graph above mean values refer to line differences: ^*^P < 0.05; ^***^P < 0.001.

The piglet probability of stillbirth and the probability of death in the first 2 days of life were analyzed in each line following the methodology described by Canario et al. (2006a). The probability was assumed to follow a binomial distribution. The factors of variation considered in the model were the effect of parity and behavioral traits as covariates. Estimates were obtained from generalized linear model of the GENMOD procedure. Both behaviors during the first hour (peak of activity) and the first 4 h (larger pattern where most of the sow activity, at least in postural changes, takes place; Figures 2–**4**), were considered as explanatory variables for the risk of mortality. The sow behavior was defined as mean duration per hour, except postural changes that was defined as a frequency per hour and responsiveness as a probability per hour. These analyses allowed the contribution of each effect to the variance reduction to be evaluated and quantified with the coefficient of determination of Hosmer and Lemeshow (1989). This coefficient of deviance reduction (DR) was established using deviance differences between successive models where explanatory variables were added one by one. The level of significance of each effect was estimated according to a likelihood ratio test.

## Results

### Farrowing process and piglet mortality

The distribution for time of onset of farrowing in old-type and modern-type sows is shown on Figure 2. The modern-type sows started farrowing more often out of the staff working hours (8–12 a.m. and 2–5 p.m.) than the old-type sows (probability of 0.66 vs. 0.46, respectively, χ^2^ = 4.23, *P* = 0.04). There was no line × parity interaction on this trait. In the global population, the effect of parity on the number of stillbirths and deaths in the first 2 days was not significant. Stillbirths were more numerous in modern-type litters than in old-type litters: the genetic trend was ΔG = +1.34 (SEΔG 0.6) stillborn piglets per litter. The number of piglets born in global, i.e., including mummified and macerated piglets was higher at second parity in modern-type litters than in old-type litters (12.3 vs. 14.6 piglets born; ΔG = +4.6 (SEΔG 2.1); *P* = 0.04). No difference was detected on that trait at first parity [12.2 vs. 12.4 piglets born; ΔG = +0.4 (SEΔG 2.2)]. Savaging piglets accounted for only one death in each line. On average 0.85 and 1.12 born alive piglets died per litter in the first 2 days in old-type and modern-type sows, respectively (ΔG = +0.54; SEΔG = 2.76; *P* = 0.43), accounting for 6.9 and 8.5% of mortality in old-type and modern-type litters (ΔG = +3.2; SEΔG 2.7; *P* = 0.51).

### Sow global activity

Sow postural activity in the first 6 h after the onset of farrowing is depicted on Figure 2. Three old-type sows vs. 1 modern-type sow were totally inactive on this period of time. The interaction between line and parity tended to be significant for almost all postural traits on this 6 h frame (*P* < 0.15), so that trends are depicted per line and parity (Table 3). Over the 6 h period, sows spent most of the time lying (>90% of time) but the first-parity modern-type sows tended to be less agitated than their old-type counterparts. Similarly, the first-parity modern-type sows also spent less time sitting than their old-type counterparts. At second parity, sow activity was globally lower and equivalent in the two lines (46 vs. 50 min; *P* = 0.60) but the modern-type sows spent less time standing and tended to spend more time lying ventrally than their old-type counterparts. Old-type sows spent more time lying ventrally and sitting, and changed of postures more frequently at first parity than at second parity. Accordingly, the relative amount of time spent lying laterally increased in old-type sows (73 vs. 89% at first and second parity, respectively) but not in modern-type sows (83 vs. 88%).

**Table 3:** **Genetic trends for sow postural activity at farrowing**.

**Trait (criterion)**	**Parity**	**Old-type sows^a^**		**Modern-type sows**		**ΔG (SEΔG)^b^**	**Pr > |t| H0: ΔG = 0^c^**
**IN THE FIRST 6 H AFTER ONSET OF FARROWING**							
Standing (min)	1	15.9		9.5		−12.8 (3.9)	0.44
	2	19.2		5.1		−28.2 (3.2)	0.004
Sitting (min)	1	19.4	|^**^^d^	10.3		−18.2 (2.7)	0.05
	2	7.4		11.1		+7.4 (2.7)	0.17
Lying ventrally (min)	1	63.8	|^**^	41.5		−44.6 (3.3)	0.39
	2	13.1		24.9		+23.6 (2.9)	0.09
Lying laterally (min)	1	265.0	|^*^	293.4		+56.8 (2.2)	0.16
	2	313.8		309.7		−8.2 (2.1)	0.80
Postural changes (#)	1	85	|^**^	62		−46 (2.4)	0.11
	2	41		42		+2 (2.5)	0.94
**IN THE FIRST HOUR AFTER ONSET OF FARROWING**							
Standing (min)	1	6.0		5.2		−1.6 (2.8)	0.69
	2	4.0		3.3		−1.4 (3.2)	0.66
Standing (#)	1	1		1	|^**^	+0 (2.9)	0.32
	2	2		0		−4 (3.7)	0.01
Sitting (min)	1	1.4		4.1		+5.4 (4.6)	0.18
	2	2.6		1.8		−1.6 (3.7)	0.58
Sitting (#)	1	6		5		−2 (2.5)	0.34
	2	4		3		−2 (2.8)	0.38
Lying ventrally (min)	1	9.5	|^*^	13.5	|^◦^	+8 (2.9)	0.27
	2	3.2		5.5		+4.6 (3.3)	0.28
Lying laterally (min)	1	37.8		33.	|^*^	−9.6 (2.3)	0.24
	2	45.4		50.0		+9.2 (2.3)	0.45
Postural changes (#)	1	16		14	|^*^	−4 (2.4)	0.40
	2	9		7		−4 (2.8)	0.35

a *Least square means*.

b *Genetic trend estimated from 1977 to 1998: ΔG = *2* × (modern-type mean—old-type mean) and SEΔG = *2* × SE (modern-type mean—old-type mean)*.

c *Probability associated with the null hypothesis (H0): ΔG = 0 (P-value)*.

d Differences between parity 1 and parity 2. Level of significance:

◦ P < 0.10;

* P < *0.05*;

** *P < 0.01*.

The line differences were mainly observed in the first 3 h after the onset of farrowing. Postural activity drastically decreased with time, and lying laterally became the main position (from the 3rd h, more than 80% of each hour was spent in this posture) with a maximum reached during the 4th h (Figure 3). Estimates of sow postural activity in the first hour are given at the bottom part of Table 3. The line × parity interaction tended to be significant for the occurrence of standing position (*P* = 0.06). A significant effect of parity was detected in modern-type sows which stood less frequently, spent even more time lying laterally and were less agitated at second parity than at first parity during the first hour.

Due to the low occurrence of sow exploratory behavior, the line × parity interaction was not estimable. The two lines differed in the time spent rooting in the first 6 h and the difference was important at second parity (11.4 vs. 2.4 min in old-type and modern-type sows, respectively; Table 4). The old-type sows performed more rooting than the modern-type sows in the first 3 h. A drop in this activity was observed in the 4th hour (Figure 4).

**Table 4:** **Genetic trends for sow exploratory behavior**.

**Trait (criterion)**	**Parity**	**Old-type sows^a^**	**Modern-type sows**	**ΔG (SEΔG)^b^**	**Pr > |t| H0: ΔG = 0^c^**
Rooting (#)	1	8	6	−4 (3)	0.46
	2	7	2	−10 (3)	0.0004
Rooting (min)	1	10.3	4.8	−11.0 (3.5)	0.16
	2	11.4	2.4	−18.0 (3.3)	0.001

a *Least square means estimated from data analyzed during the first 6 h after onset of farrowing*.

b *Genetic trend estimated from 1977 to 1998: ΔG = *2* × (modern-type mean—old-type mean) and SEΔG = *2* × SE (modern-type mean—old-type mean)*.

c *Probability associated with the null hypothesis (H0): ΔG = 0 (P-value)*.

### Maternal behavior

**Figure 5:**
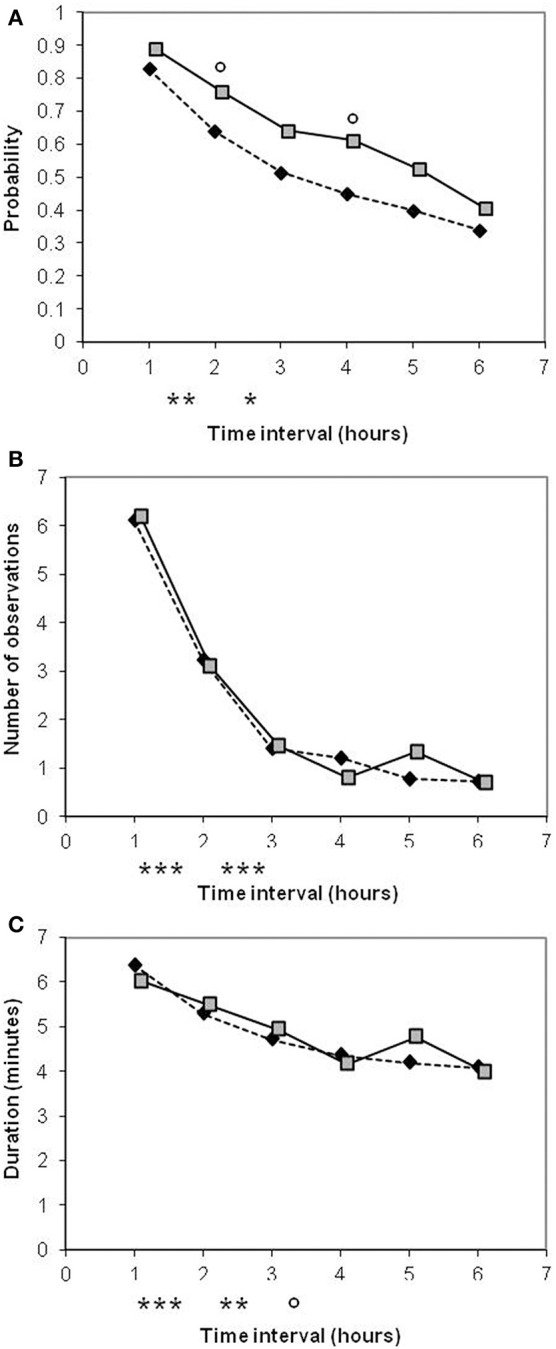
**Comparison of old-type sows (♦) and modern-type sows (**
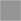
**) for maternal responsiveness (A) and maternal attentiveness occurence (B) and duration (C) during the first 6 h after the onset of farrowing**. Significance levels below the X axis refer to differences between successive periods of time, obtained with both lines merged together. Significance levels reported on the graph above mean values refer to line differences: ^◦^*P* < 0.10; ^*^P < 0.05; ^**^P < 0.01; ^***^P < 0.001.

Estimates of sow maternal behavior are shown in Table 5. There was a significant line × parity interaction for responsiveness toward progeny when defined as occurrence, and a tendency for attentiveness when defined as duration. The probability of response to a nose contact differed between lines at second parity in favor of modern-type sows. The first-parity old-type sows spent more time watching their piglets than their second-parity and modern-type counterparts. At second parity, differences disappeared and sows from both lines spent less time in attention toward their progeny than in first parity. Responsiveness decreased with time, but modern-type sows remained more responsive than their old-type counterparts (Figure 5). Attention also decreased with progress of farrowing. As regards to sow reactivity at first manipulation of a newborn by human, no piglet was screaming during handling. Sow's maximum posture reached was similar in the two lines: most of the sows remained in lateral position (33/36 and 30/34 in old-type and modern-type sows, respectively). However, at second parity, even if not significant, the modern-type sows tended to perform more grunts in reaction to piglet handling than their old-type counterparts (*P* = 0.19). The line × parity interaction approached significance (*P* = 0.15) and a tendency for greater vocal reaction at second parity than at first parity was observed in both lines (*P* < 0.10). The maximum posture achieved at the first nose contact between the sow and a newborn piglet did not differ. Approximately two third of the sows in each line were moving to a position different from lying laterally (15/27 and 25/36 in old-type and modern-type sows, respectively). The average vocal reaction was also equivalent between the lines (but 9/27 old-type and 22/36 modern-type sows did not grunt at all). In addition, the modern-type sows tended to react less with postural change and more with vocalizations from first to second parity. Conversely, the probability of aggressive reaction to the first newborn piglet approached significance: the modern-type sows tended to have a gentler reaction than old-type sows at first parity.

**Table 5:** **Genetic trends for sow maternal behavior**.

**Trait (criterion)**	**Parity**	**Old-type sows^a^**		**Modern-type sows**		**ΔG (SEΔG)^b^**	**Pr > |t| H0: ΔG = 0^c^**
**VIDEO OBSERVATION SOW REACTION IN THE FIRST 6 h AFTER ONSET OF FARROWING**							
Piglet responsiveness (*p*)	1	0.67	|^***^^e^	0.72		+0.10 (1.09)	0.21
	2	0.48		0.64		+0.32 (1.13)	0.01
Piglet attentiveness (#)	1	30	|^***^	18		−24 (2.59)	0.04
	2	6		10		+8 (2.73)	0.08
Piglet attentiveness (min)	1	37.9	|^***^	17.7		−40.4 (0.07)	0.003
	2	9.7		13.3		+ 7.2 (0.05)	0.37
**DIRECT OBSERVATION AT FARROWING ONSET**							
**Reaction to first piglet handling (*N* = 36 and *N* = 34)**							
Maximum posture reached (class)^d^	1 + 2	0.14		0.12		−0.04	0.88
Vocalizations (class)	1	0.15	|^◦^	0.11	|^◦^	−0.08 (6.44)	0.74
	2	0.52		0.77		+0.50 (2.6)	0.19
**Reaction to first nose contact with a piglet (*N* = 27 and *N* = 36)**							
Maximum posture reached (class)	1	1.39		1.43	|^◦^	−0.26 (3.83)	0.92
	2	1.15		0.79		−0.72 (2.92)	0.39
Vocalizations (class)	1	1.19		0.66	|^◦^	−1.06 (3.23)	0.21
	2	0.93		1.23		+0.60 (2.92)	0.46
Sniffing piglet (*p*)^d^	1 + 2	0.75		0.94		+0.38	0.19
Aggressive reaction (*p*)	1	0.60		0.51		−0.18	0.11
	2	0.52		0.50		−0.04	0.45

a *Least square means*.

b *Genetic trend estimated from 1977 to 1998: ΔG = 2 × (modern-type mean—old-type mean) and SEΔG = 2 × SE (modern-type mean–old-type mean)*.

c *Probability associated with the null hypothesis (H0): ΔG = 0 (P-value)*.

d *Due to low sample size and low occurrence of the trait, the line × parity interaction could not be estimated*.

e *Differences between parity 1 and parity 2. The sign for significance is attributed to the largest value*.

◦ P < 0.10;

*** *P < 0.001*.

### Newborn piglet behavior

Results of newborn piglet behavior at birth are shown in Table 6. The line × parity interaction was significant for mobility and vocalizations when put in a new environment (*P* < 0.01) and a tendency was obtained for respiratory difficulty (*P* = 0.14). Second-parity modern-type piglets showed higher difficulties to breath at birth than their old-type counterparts. At both first and second parities, modern-type piglets had a lower vitality than old-type piglets. Time to reach the udder and to the first colostrum intake were higher in modern-type than old-type piglets.

**Table 6:** **Genetic trends for newborn piglet behavior**.

**Trait (criterion)**	**Parity**	**Old-type sows ^a^**	**Modern-type sows**	**ΔG (SEΔG)^b^**	**Pr > |t| H0: ΔG = 0^c^**
**VIDEO OBSERVATION**					
Time to first udder contact (min)^d^	1 + 2	43 (6)	57 (6)	+28	0.14
Time to first colostrum intake (min)^d^	1 + 2	69 (8)	86 (7)	+34	0.16
**DIRECT OBSERVATION**					
Respiratory difficulty (*p*)	1	0.03	0.04	+0.02 (1.25)	0.77
	2	0.03	0.07	+0.08 (1.19)	0.01
Mobility at birth (class)	1	1.40	1.20	−0.40 (2.20)	0.02
	2	1.50	1.23	−0.54 (2.20)	0.003
Vocalizations at birth (class)	1	0.82	0.46	−0.72 (2.51)	0.01
	2	0.64	0.27	−0.74 (2.55)	0.0004

a *Least square means. N = 506 vs. 477 for respiratory difficulty; N = 508 vs. 488 for mobility; and N = 500 vs. N = 475 for Vocalizations at birth in old-type and modern-type lines, respectively*.

b *Genetic trend estimated from 1977 to 1998: ΔG = 2 × (modern-type mean—old-type mean) and SEΔG = 2 × SE (modern-type mean—old-type mean)*.

c *Probability associated with the null hypothesis (H0): ΔG = 0 (P-value)*.

d *Due to low sample size and low occurrence of the trait, the line × parity interaction could not be estimated*.

### Relationship between piglet mortality and sow and piglet behavior

Estimates of the probability of stillbirth and death are shown in Tables 7, 8, respectively.

**Table 7:** **Association between probability of stillbirth and sow behavior**.

**Sows**	**Old-type piglets**			**Modern-type piglets**		
**Model**	**Sign^a^**	**D**	**DR (%)^b^**	**Sign**	**D**	**DR (%)**
**IN THE FIRST HOUR OF LACTATION *N* = 934**						
(0) = intercept	+	279.26		+	296.98	
(1) = (0) + Parity	+	276.92	0.84	−	294.3	0.90
(2) = (1) + Postural changes	−	273.88	1.10	+	287.68	2.25^◦^
(3) = (2) + Standing	−	271.54	0.85	−	282.66	1.75
(4) = (3) + Sitting	−	271.22	0.12	−	282.54	0.04
(5) = (4) + Lying ventrally	−	262.22	3.32^**^	−	278.84	1.31
(6) = (5) + Lying laterally	−	259.36	1.09	−	253.48	9.10^***^
**IN THE FIRST 4 H OF LACTATION *N* = 583**						
(0) = intercept	−	125.15		+	168.68	
(2) = (1) + Parity	−	123.72	1.14	+	166.70	1.17
(3) = (2) + Postural Changes	−	123.61	0.09	+	166.56	0.08
(4) = (3) + Standing	+	117.95	4.58^**^	+	161.62	2.97^*^
(5) = (4) + Sitting	+	117.94	0.01	+	161.25	0.23
(6) = (5) + Lying ventrally	−	117.55	0.33	+	158.50	1.71
(7) = (6) + Lying laterally	+	117.48	0.06	+	151.20	4.61^*^
(8) = (7) + Rooting	−	117.42	0.05	−	144.13	4.68^**^
(9) = (8) + Piglet responsiveness	+	117.06	0.31	+	144.01	0.08
(10) = (9) + Piglet attention	−	101.62	4.67^**^	−	137.36	4.62^*^

*Reduction of deviance due to the addition of behavioral traits as explanatory variables*.

a *Sign of the corresponding estimate indicates positive or negative association between stillbirth probability and the explanatory variable. D, deviance; DR, deviance reduction*.

b Level of significance according to Likelihood Ratio Test (LRT) statistics. Level of significance:

◦ P < 0.10;

* P < 0.05;

** P < 0.01;

*** *P < 0.001*.

**Table 8:** **Association between probability of death in the first 2 days after birth and sow and piglet behavior**.

**Model**	**Old-type piglets**			**Modern-type piglets**		
	**Sign^b^**	**D**	**DR (%)^c^**	**Sign**	**D**	**DR (%)**
**CONTINUOUS SOW BEHAVIOR IN THE FIRST HOUR OF LACTATION (VIDEO) *N* = 847**						
(0) = intercept	−	253.02		−	256.62	
(1) = (0) + Parity	+	252.72	0.12	−	256.58	0.02
(2) = (1) + Postural changes	+	252.46	0.10	+	254.98	0.62
(3) = (2) + Standing	+	249.12	1.32	+	252.42	1.00
(4) = (3) + Sitting	+	249.12	0.00	−	252.16	0.10
(5) = (4) + Lying ventrally	+	248.92	0.08	−	250.8	0.54
(6) = (5) + Lying laterally	+	248.18	0.30	+	247.28	1.40
**IN THE FIRST 4 H OF LACTATION *N* = **370****						
(0) = intercept	+	108.90		+	99.18	
(2) = (1) + Parity	+	108.46	0.40	+	99.18	0.00
(3) = (2) + Postural changes	−	107.92	0.50	−	96.64	2.56^*^
(4) = (3) + Standing	−	107.88	0.04	−	91.32	5.51^**^
(5) = (4) + Sitting	−	99.72	7.56^**^	−	89.22	2.30^*^
(6) = (5) + Lying ventrally	−	99.04	0.68	−	88.74	0.54
(7) = (6) + Lying laterally	−	98.8	0.24	−	87.8	1.06
(8) = (7) + Rooting	+	98.52	0.28	−	87.74	0.07
(9) = (8) + Piglet responsiveness	−	97.24	1.30	−	87.48	0.30
(10) = (8) + Piglet attention	−	89.58	9.07^***^	−	87.68	0.07
**OBSERVED MATERNAL BEHAVIOR *N* = **398****						
(0) = intercept	−	112.38		−	133.84	
(1) = (0) + Parity	−	110.36	1.78	−	133.84	0.00
(2) = (1) + H_Max posture reached^a^	+	110.28	0.07	+	127.64	4.63^*^
(3) = (2) + H_Vocalization	−	107.42	2.59^◦^	−	127.64	0.00
(4) = (3) + C_Max posture reached	−	107.26	0.15	+	127.42	0.17
(5) = (4) + C_Vocalization	+	107.1	0.15	+	127.28	0.11
(6) = (5) + C_Sniffing	+	106.06	0.97	+	122.94	3.41^◦^
(7) = (6) + C_Aggressive reaction	−	104.52	1.45	+	121.08	1.51
**NEWBORN PIGLET BEHAVIOR *N* = **990****						
(0) = intercept	−	283.62		−	322.96	
(1) = (0) + Parity	−	283.34	0.10	−	322.72	0.07
(2) = (1) + Respiratory difficulties	+	274.68	3.06^◦^	+	313.9	2.73^*^
(3) = (2) + Mobility at birth	−	268.34	2.31^*^	−	310.2	1.18^◦^
(4) = (3) + Vocalizations at birth	+	268.04	0.11	−	309.86	0.11

*Reduction of deviance due to the addition of behavioral traits as explanatory variables*.

a *H, handling piglet reaction; C, nose contact reaction*.

b *Sign of the corresponding estimate indicates positive or negative association between stillbirth probability and the explanatory variable. D, deviance; DR, deviance reduction*.

c Level of significance according to Likelihood Ratio Test (LRT) statistics. Level of significance:

◦ P < 0.10;

* P < 0.05;

** P < 0.01;

*** *P < 0.001*.

Regarding the probability of stillbirth, the trait associated with the largest effects, i.e., time spent lying laterally during the first hour, explained 1.1 and 9.1% of DR in old-type and modern-type sows, respectively. In the first hour, time spent lying ventrally was also negatively associated with the probability of stillbirth in old-type sows (3.3% DR). Moreover, the probability of stillbirth tended to increase in piglets born from modern-type sows that realized more postural changes (2.2% DR). In the first 4 h, time spent standing was a factor of stillbirth in both lines (4.6 and 3.0% DR in old-type and modern-type sows, respectively). Sows more attentive toward their piglets had a lower probability of stillbirth in the two lines (DR of 4.7 and 4.6%). Rooting was associated with a consistent DR in the modern-type sows (4.7%). Lying laterally was a risk factor in modern-type sows (4.6% DR). The probability of stillbirth decreased with rooting and increased with lying laterally in modern-type sows. The influence of other behavioral traits on the probability of stillbirth was less than 2%.

Due to the relatively low occurrence of crushing and starvation, the two causes of mortality were not distinguished. The probability of death in the first 2 days was lowly influenced by sow behavior in the first hour but varied with the duration of standing and sitting, and the number of postural changes in the first 4 h in modern-type sows (more activity—lower risk of piglet death; 2.3–5.5% DR). In old-type sows, the probability of death decreased with sitting activity (7.6% DR) and attention to piglets (9.1% DR). The duration of lying laterally was not a factor of variation for the risk of death in any of the two lines. Sow reaction at the onset of farrowing affected the probability of death: the maximum posture reached in response to piglet handling explained 4.6% of DR in modern-type sows (more reaction—higher risk of death) and vocalizations explained 2.6% of DR in old-type sows (more grunts—lower risk of death). The probability of mortality tended to increase with sniffing of the first newborn piglet in modern-type sows (3.4% DR). Both old-type and modern-type piglets with greater respiratory difficulties at birth were more susceptible to die in the first 2 days (3.1 and 2.7% DR in old-type and modern-type piglets, respectively). Mobility at birth explained a greater part of DR in old-type piglets than in modern-type piglets (2.3 vs. 1.2% DR). These analyses showed higher contributions of sow behavior than piglet behavior to the probability of mortality.

## Discussion

### General considerations

Swine behavior and welfare can be affected by genetic selection due to genetic correlations with the traits included in the breeding goal. Some genetic correlations may be antagonistic, so that the modifications in behavior may be limited. Selection on litter size has reduced the selection pressure on the formerly selected traits. The statistical power of the experimental design available to address behavior changes over a 21-year selection period was limited by the rather low number of animals available and the large within-line variability of behavioral responses classically reported in the literature (e.g., Koolhaas et al., 1997; Wechsler and Hegglin, 1997). Nevertheless, the power was sufficient to detect several significant differences between lines that demonstrate that selection has modified both sow farrowing activities and piglet vitality at birth.

The behavioral response also depends on conditional factors. It may be relatively less intense when observations are performed in the home environment rather than in a novel environment. In addition, sows from the two lines were placed in adjacent crates alternating old-type and modern-type sows, which tended to homogenize results between the 2 lines with the progress of lactation. However, farrowing ought to be a process experienced uniquely by each individual. We therefore expected a substantial variation in behavioral traits during this critical period that causes high acute stress in the sow and represents a challenge for newborns who must adapt quickly to extra-uterine life (Nowak et al., 2000). Human intervention was limited in order to study the biological phenomenon as objectively as possible and to evaluate the capacity of the sow to produce piglets.

Our results are in accordance with previous estimates obtained for the 2nd generation populations, in which the total number of piglets born per litter did not significantly differ between old-type sows (11.9 ± 0.5) and modern-type sows (12.7 ± 0.5) but an increase of stillbirths had been detected: +1.34 stillborn piglets per litter and +8.4% of stillbirths on average (Canario et al., 2007a). The reason for the lack of difference in litter size between the lines at both first and second parities was discussed: prenatal losses and intra-uterine crowding during late gestation were more severe in modern-type sows. The sow populations compared in the present study differed as regards to backfat depth but not in body weight at farrowing, and fatness was a large determinant of stillbirth in modern-type sows (20.3% of DR for the probability of stillbirth; Canario et al., 2007a).

In the present study, early piglet mortality was not extremely high (7 and 8.5% in old-type and modern-type sows, respectively), but substantial losses were observed previously more than 48 h after the beginning of lactation (Canario, 2006). All born alive piglets were kept under the sow, whatever the litter size. In the literature, litters with more numerous stillborn piglets have been shown to face higher pre-weaning mortality (Leenhouwers et al., 1999; Casellas et al., 2004). Accordingly, in modern-type sows, on average one piglet was lost per litter in the 2 days after farrowing. The fact that sows were maintained between fences might have prevented them from displaying the whole range of their behaviors, so genetic trends and the role of dams on the survival of their progeny might have been underestimated. Behavioral results will be discussed in connection with the welfare issues related to selective breeding, the discussion being facilitated by our results revealing the relationships with the risk of piglet mortality.

### Genetic trends in the global activity of parturient sows

A major rule of animal welfare is to limit pain by prevention or treatment. In conventional farming, farrowing events are routinely assisted with use of ocytocin injection to stimulate contractions and vaginal palpations to release piglets that might be blocked in the vaginal canal. In our study, human interventions at farrowing were limited to cases of extreme necessity, involving survival of piglets and in such cases, the litter was not included in analyses. In past years, several reports have suspected an increase in the duration of farrowing with selection for litter size (Rutherford et al., 2013). Although expected due to the genetic variation in this trait (*h*^2^ < 0.10) and its genetic association with stillbirth (Holm et al., 2004; Canario et al., 2006b), such a trend could not be detected distinctly even in the current low-interventionist design. In global populations, even though modern-type piglets were heavier at birth (+260 g on average compared with old-type piglets), we found no distinct trend in farrowing kinetics between old-type and modern-type first-parity sows and a non-significant average increase of 0.8 h of farrowing in second-parity sows with a risk of stillbirth that increased strongly with the time elapsed from the onset of farrowing in modern-type piglets (Canario et al., 2007a).

Sows prepare farrowing several hours in advance and their activity decreases with impending parturition (e.g., Jensen, 1993). Interestingly, our results suggest that modern-type sows can postpone the onset of farrowing so as to avoid human presence. If so, this represents a fairly significant adaptation to their environment. This reaction can be interpreted as increased anxiousness of modern-type sows, which have a higher stillbirth rate when farrowing occurs during the presence of staff (Hemsworth et al., 1999; Janczak et al., 2003). Grandinson et al. (2003) found no phenotypic relationship between avoidance of humans and piglet mortality during early lactation, but a positive genetic association in favor of selection against this behavior.

At farrowing, lower reactivity facilitates the continuity of the process and thus limits birth difficulties. Lying laterally can be considered as a good behavior (Thodberg, 2001). A limited time spent in the lying posture indicates difficulties in coping with this critical event. But on the contrary, total inactivity is also indicative of farrowing difficulties. This assumption was confirmed by a positive association between the time spent lying during the first hour and the risk of stillbirth in modern-type sows. Also, Engelsma et al. (2011) estimated favorable but not significant correlations between the sows' genetic potential for piglet production and calmness around farrowing (less postural changes and lower activity). The higher contribution of the lateral lying posture to the risk of stillbirth in modern-type sows indicates that they experience greater uterine and maternal fatigues that lead to dystocia than old-type sows. The same inactivity has been observed in mice selected for lean-tissue growth rate (McPhee et al., 2001). More generally, the first 3 h after the onset of farrowing are a highly sensitive period during which the sow must adapt to motherhood.

The risks of stillbirth and neonatal death are reported to undergo little variation during the first parities (e.g., Arango et al., 2006) although Canario et al. (2006a) found a decrease in stillbirth from first to second parity in the French Large White dam population. In the study lines, the effect of parity on stillbirth was not significant (Canario et al., 2007a). Stillbirth in young sows might be related to insufficient size of the birth canal (Pejsak, 1984), especially in modern-type sows that produce heavier piglets. As regards to behavior, sows acquire maternal experience at the first parity. Multiparous sows display faster and easier behavioral adaptation than primiparous sows due to a lower susceptibility to the stress of farrowing, leading to lower reactivity (Thodberg, 2001). Such a difference due to maternal experience was detected only in old-type sows that spent more time lying laterally at second parity than at first parity during the 6-h observation period. In modern-type sows, the level of total inactivity was very high at both first and second parities, presumably in relation with the higher incidence of prenatal deaths and the higher mean piglet weight found in these sows compared with old-type sows (Canario et al., 2007a).

Furthermore, we found that modern-type sows were on average less active than old-type sows, which is in line with McPhee et al. (2001) who found that sows selected for lean-tissue growth rate are less active at farrowing. Genetic trends toward decreased time spent sitting (−18 min/6 h) and changing of posture (−46 postural changes/6 h) in first-parity sows and decreased standing activity in second-parity sows (−28 min/6 h and −4 times during the first hour) were estimated. Sitting can indicate stress (Dybkjaer, 1992) and possibly the sow's motivation but inability to perform nest-building activities when blocked in a crate (Hartsock and Barczewski, 1997; Jarvis et al., 2001; Thodberg, 2001). This posture was not observed beyond the 3rd hour after the onset of farrowing. The old-type first parity sows spent twice more time sitting than second parity sows, who spent more time standing and rooting. On the other hand, sitting is an ideal posture for observing piglets from a distance, as an alternative to closer contact on the ground if newborns are source of anxiety. The higher occurrence of sitting in primiparous sows confirms such a hypothesis. Standing is the more extreme postural change at farrowing. This posture is reached for maintenance activities and ground-directed activity (*r* = 0.96 and 0.86 between standing and ground-directed activity in the first 6 h in old-type and modern-type sows, respectively; *P* < 0.0001 in both cases). Also, standing allows the sow to establish motivated contacts with newborns. If this posture is not associated with the risk of stillbirth in the first hour, it becomes a substantial explanatory variable later in the farrowing process. Standing most likely favor pauses in the farrowing process and risk of hypoxia if it causes early rupture of the umbilical cord of unborn piglets. Consequently, the genetic trend toward less standing activity is positive for piglet welfare. In our study, the weight of old-type and modern-type sows did not differ at farrowing, but the modern-type sows produced heavier litters (Canario et al., 2007a). In addition, the higher frequency of postural changes in first-parity old-type sows reflects restlessness elicited by novelty (Cronin et al., 1993). The effect of parity on this trait was previously reported by Li and Gonyou in gestating sows (Li and Gonyou, 2007).

A genetic trend toward more time spent lying ventrally was observed in second-parity sows (+24 min/6 h; *P* = 0.09). Lying ventrally can reveal a discomfort as compared to lying laterally at farrowing. This posture facilitates the observation of the environment and newborns. It can therefore be important for sow-progeny bonding, as shown by the negative association between this behavior and stillbirth observed in old-type sows during the first hour. The time spent lying ventrally was higher in first- than second-parity old-type sows, thus revealing that, like sitting, it also reflects a reaction to novelty. The modern-type second parity sows may use this posture for bonding or as a compromise if farrowing is so painful or laborious that they are unable to reach a higher posture. Normally, pain should be reduced naturally via the analgesia mediated by opioids released at farrowing (Jarvis et al., 1999). Lying ventrally also means that the sows voluntarily hide their udder which complicates the first intake of colostrum. However, the observation of this posture at farrowing was not associated with a risk of death in newborn piglets.

Nesting is a sow activity that is extremely robust to domestication. It is performed even in absence of nesting materials through rooting, i.e., ground-directed activity (Jensen, 2002). When performed at farrowing, its occurrence declines rapidly with the release of oxytocin (Vestergaard and Hansen, 1984; Castrén et al., 1993), but it is sometimes claimed to be an inappropriate activity because sows are restless while giving birth (Jensen, 1993; Thodberg et al., 1999; Damm et al., 2000). Jensen (2001) suggested that rooting might continue in parturient sows that experience stress until sufficient feedback is obtained and the sow returns to homoeostasis. Also, such continuation of the activity can reflect the perception of an unsatisfactory nest environment and the willingness to improve it (Cronin et al., 1993). We found a genetic trend for decreased rooting activity in second-parity sows. However, this discrepancy between old-type and modern-type sows might merely find explanation in the greater farrowing difficulties of modern-type sows. In the present experiment, sows were supplied with a limited amount of straw. As such, rooting can be interpreted as a clue for good maternal behavior. In agreement, the risk of stillbirth increased with low rooting in modern-type sows. Genetic variability in rooting does exist: Meishan sows, often referred to as sows with a good mothering style (calm temperament), spend more time manipulating straw and rooting at farrowing than Large White sows when raised in individual pens (Rydhmer and Canario, 2014). In line with these observations, Rauw (2001) found that selected females spent less time in floor nosing activity when comparing a mice line selected for litter size with the control line.

### Genetic trends in the maternal behavior of parturient sows

The maternal behavior of sows is elicited at farrowing and is expressed through interactions with the newborns, leading to a stable relationship between the mother and her progeny. The results obtained here on maternal behavior differ according to parity. They will be discussed mainly in relation with the risk of early death. Sows often stand, turn and sniff the first piglets born (Jensen, 1986), and this behavior declines as more piglets are farrowed (Johnson and Marchant-Forde, 2009). The first-parity old-type sows tended to display a stronger reaction. But only the behavior “sniffing by the sow” was explicative of a higher risk of death in the first 2 days in modern-type piglets. The probability of aggressive reaction decreased by 18% of genetic trend in first parity sows. These elements indicate a discomfort and lesser adaptation of old-type sows to their farming conditions at farrowing. But again, the prostrate attitude of modern-type sows, although expected to be more excitable animals, may prevent fierce reactions to their newborns, especially since they are blocked in a crate.

After completion of farrowing, sows are inactive for more than 90% of the time during the first 48 h, which is an adaptive response that reduces piglet crushing (Johnson and Marchant-Forde, 2009). Early restlessness and responsiveness to piglets are correlated at the onset of farrowing (*r* = 0.63 and 0.51 in old-type and modern-type sows, respectively; *P* < 0.05). According to the literature, this behavior is correlated with the risk of crushing piglets (Wechsler and Hegglin, 1997; Damm et al., 2005). However, in the present study, postural activity within the first hours of lactation was positively associated with piglet survival in the modern-type sows only. In line with this, higher activity at farrowing may predict the sows' ability to react to piglets that become trapped when the sow lies down. Thodberg et al. (2002) found that gilts that were active during farrowing continued being so the next day. Further investigation of behavior in the first days of lactation is required to evaluate the relationships with crushing. Grandinson et al. (2003) showed that the heritability of the sow's postural reaction to a screaming piglet on the farrowing day is low and tends to be negatively correlated with mortality at the genetic level.

McPhee et al. (2001) highlighted that sows selected for high lean-tissue growth rate are more responsive to piglets than sows selected for low lean-tissue growth rate. In our experiment, a positive genetic trend toward higher responsiveness to nose contact initiated by piglets was observed in second-parity sows (probability of +32%/6 h). This trend was explained by the decreased reactivity to piglets with parity in old-type sows, while modern-type sows maintained a high reaction at both first and second parities. Pedersen et al. (2003) suggested that sow responsiveness to newborn piglets is an indicator of good maternal care. At the genetic level, Grandinson et al. (2003) found a positive association between sow responsiveness to piglets and piglet survival in a modern-type population whereas in the present study, we found weak relations between the two traits. It is also possible that such maternal behavior helps to reduce anxiety. Indeed, Lonstein (2005) emphasized that in rats, dam-pup interactions contribute to reducing anxiety during early lactation. As a consequence, modern-type sows may be more anxious and express their anxiety behaviorally. This idea is supported by observations by Grandin and Dessing (2014) who report a more excitable temperament in pigs selected for lean growth, resulting in animals more reactive to sudden novelty. Selection for lean growth may have increased sensitivity to stress in the French Large White population, due to the negative association between lean growth and cortisol production (Mormède et al., 2011). Significant modifications of the stress-responsive systems were observed in the 2nd generation of the experiment: modern-type pigs had lower corticosteroid levels than old-type pigs (Foury et al., 2009).

An association between a sow's attention toward her newborn piglets and maternal ability was clearly established. A genetic trend toward lower attention was found in first-parity sows (−24 times and −40 min/6 h). Attention was positively associated with survival at birth in both lines and survival during the first 2 days after birth in the old-type line. This maternal behavior was not associated with a certain posture (*r* < 0.32 with the time spent in different postures in the two lines; results not shown). A more direct and easy measurement of maternal willingness is the reaction to handling of the newborns by humans. In the present study, humans were often present around the farrowing crate in the days preceding farrowing and manipulated newborns at farrowing. Although no genetic trend was found, vocalizations were related to a lower piglet death rate in old-type sows. Conversely, the intensity of vocalizations was not related to a risk of mortality in modern-type sows. Few investigations of postural reactions to the handling of newborn piglets by humans have been reported in genetic studies. Grandinson et al. (2003) found no genetic variation in the first days after farrowing, probably due to the way the test was performed.

### Genetic trends in the behavior of newborn piglets

Piglets play a major role in their own survival after birth, and it depends largely upon the quality of their interactions with the dam. Early survival depends on both the ability of the sow to produce colostrum and her nursing behavior, as well as on the ability of each piglet to acquire a sufficient quantity of colostrum (Le Dividich et al., 2005). To their advantage, piglets are behaviorally precocious, with coordinated locomotion from birth, but they are not assisted by the sow in their teat-seeking activity (Nowak et al., 2000). Modern-type newborn piglets suffered from the greater farrowing difficulties of their dam, even more so at second than at first parity. In addition to an increased rate of stillbirth, they displayed greater respiratory difficulties due to hypoxia than old-type piglets. The modern-type piglets born later in the farrowing process were at a higher risk of stillbirth than their old-type counterparts (Canario et al., 2007a). Piglets having suffered from hypoxia at birth do not necessarily die during the birth process, but show lower vitality after birth and then lye still until they recover. If they survive, they often remain too weak to be able to suckle efficiently (Herpin et al., 1996). Vitality is beneficial to survival: sows with a higher genetic potential for piglet survival produce piglets that take shorter time to reach the udder and suckle (Knol et al., 2002). In the present study, modern-type newborn piglets were less mobile when placed in the weighing box and then, once back at the rear end of their farrowing dam, tended to be slower to access the udder and suckle for the first time. The proportion of piglets with low vitality *per se* (i.e., without breathing difficulties) was high. We demonstrated previously that modern-type piglets are less physiologically mature at birth than old-type piglets (Canario et al., 2007b). Significant differences in the body composition and physiological maturity of newborn piglets were also found between lean and fat genotypes (Herpin et al., 1993), suggesting that selection for leanness has affected piglet maturity at birth. This negative genetic trend has direct implications on the capacity of survival during early lactation. A similar conclusion was drawn by Leenhouwers et al. (2002) who found that piglets with a high genetic merit for survival were similar to piglets from genetically obese lines, with increased cortisol levels that allowed them to endure the stress of farrowing and face the difficulties of neonatal life.

As growth rate on day 1 was similar in old-type and modern-type litters (Canario, 2006), it may be assumed that the production of colostrum was similar in old-type and modern-type sows and as a consequence, that the colostrum intake of their piglets did not differ. However, modern-type sows spent longer time in a lying position which facilitated access to the udder for colostrum uptake at farrowing (i.e., with udder exposed). Several postural clues as to the old-type sows' reluctance to nurse their progeny were highlighted, especially at first parity. There was a trend for less time spent lying laterally with udder exposed during the first 6 h of lactation in first-parity old-type sows. In general, old-type sows also spent more time nest-building than their modern-type counterparts, which limited udder contact. Hence, the more favorable (inactive) behavior of modern-type sows would in some sense be beneficial to their low-vitality piglets. The higher losses observed in modern-type litters could also be due to the fact that weak modern-type piglets must access the udder within a shorter time in order to survive. Piglets must regulate their body temperature, an energy demanding process, during the time interval between birth and first colostrum intake, and this interval was increased by human manipulation. The lower maturity of modern-type piglets (Canario et al., 2007b) could be due partly to the slightly shorter gestation of modern-type sows (−0.7 ± 0.3 day; Canario et al., 2006a), which may result in a higher proportion of preterm farrowings that reduce the production of colostrum (by 40% when farrowing takes place on day 110–111 of gestation; Milon et al., 1983) and increase losses during early lactation (Casellas et al., 2004). There is growing evidence that it is important to consider the two sources of genetic variation, i.e., the dam and the piglet, to improve piglet traits (Leenhouwers et al., 2001).

## Conclusion

This study provides an insight into the intricate interactions between maternal and newborn behavior as determinants of piglet survival in the context of genetic trends associated with the selection for lean growth rate and prolificacy at birth. The experiment was designed to assess the capacity of sow investment in piglet production. Genetic differences were obtained for the sow's activity at farrowing and suggested a higher pressure on modern-type sows. The influence of parity was more distinct in old-type sows. Genetic trends toward higher reactivity to newborn piglets were observed and related to the substantial changes on production traits. Sow behavior at farrowing was found to contribute substantially to piglet survival. Some interesting associations to consider in breeding programs were outlined, like the relationship between lying laterally during the first hours of farrowing, a higher stillbirth rate and poorer welfare in modern-type sows. The importance of sow attention toward piglets was emphasized. Piglet survival depends on the sow's capacity to farrow and the intrinsic viability of piglets. It would be interesting to analyze mother-progeny interactions during the first week of lactation to address welfare issues related to poor vitality in modern-type piglets.

### Conflict of interest statement

The authors declare that the research was conducted in the absence of any commercial or financial relationships that could be construed as a potential conflict of interest.
